# Fusogenic Nanoreactor‐Based Detection of Extracellular Vesicle‐derived miRNAs for Diagnosing Atherosclerosis

**DOI:** 10.1002/smll.202501789

**Published:** 2025-04-21

**Authors:** Jiyoon Lee, Kiyoon Kwon, Min Ji Cho, Taesang Son, Yuna Roh, Sugi Lee, Dae‐Soo Kim, Moo‐Seung Lee, Hyun Seung Ban, Jang‐Seong Kim, Eun‐Kyung Lim, Sang‐Hak Lee, Goo Taeg Oh, Jong‐Gil Park, Tae‐Su Han

**Affiliations:** ^1^ Korea Research Institute of Bioscience and Biotechnology (KRIBB) Daejeon 34141 Republic of Korea; ^2^ KRIBB School Korea University of Science and Technology (UST) Daejeon 34113 Republic of Korea; ^3^ Division of Cardiology Department of Internal Medicine Yonsei University College of Medicine Seoul 03722 Republic of Korea; ^4^ Heart‐Immune‐Brain Network Research Center Department of Life Science and College of Natural Sciences Ewha Womans University Seoul 03760 Republic of Korea; ^5^ School of Medicine Sungkyunkwan University Suwon 16419 Republic of Korea

**Keywords:** atherosclerosis diagnosis, extracellular vesicle, fusogenic nanoreactor, microrna, rolling circle amplification

## Abstract

Extracellular vesicle (EV) microRNAs (miRNAs) are critical liquid‐biopsy biomarkers that facilitate noninvasive clinical diagnosis and disease monitoring. However, conventional methods for detecting these miRNAs require EV lysis, which is expensive, labor‐intensive, and time‐consuming. Inspired by natural viral infection mechanisms, a novel strategy is developed for detecting EV miRNAs in situ via vesicle fusion mediated by viral fusion proteins. A padlock probe encapsulated within fusogenic liposomes is activated by target miRNAs, thereby initiating a highly sensitive and specific rolling circle amplification (RCA) reaction. Three EV miRNAs associated with atherosclerosis are successfully analyzed using this method, thereby enabling clear differentiation of healthy and diseased mice at several disease stages. Overall, the developed platform offers a simple approach for detecting EV miRNAs and demonstrates significant potential for broad use in applications involving disease diagnosis and monitoring.

## Graphical abstract

This study introduces an all‐in‐one diagnostic system that amplifies target genes through interactions between serum EVs and fusogenic liposomes. Using padlock probes and enzymes within liposomes, the system enables miRNA amplification upon fusion. It successfully detects miR‐33a, miR‐126, and miR‐145, showing strong diagnostic performance comparable to RT‐qPCR and effectively distinguishing normal and atherosclerotic mice.

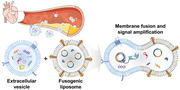

## 1 Introduction

Atherosclerosis is a chronic inflammatory disease characterized by the accumulation of lipids, inflammatory cells, and fibrous elements within arterial walls, leading to the formation of plaque. This condition is a primary underlying cause of cardiovascular diseases, including coronary artery disease and stroke, and is among the leading causes of morbidity and mortality worldwide.^[^ ^1^ ^]^ Reducing the burden of atherosclerosis and improving patient outcomes requires accurate detection and effective monitoring. Atherosclerosis is currently diagnosed using imaging modalities, such as ultrasound, computed tomography, and magnetic resonance imaging, as well as blood tests to assess lipid profiles and inflammatory markers. These methods often require expensive specialized equipment and may not be sufficiently sensitive to detect diseases at various stages.^[^ ^2^ ^]^ Liquid biopsy, a minimally invasive diagnostic approach that uses biomarkers in bodily fluids, has emerged as a promising alternative in recent years.^[^ ^3^ ^]^

Extracellular vesicles (EVs) are nanosized membrane‐bound particles secreted by various cell types that play key roles in intercellular communication by transferring bioactive molecules, including proteins, lipids, and nucleic acids. EVs are remarkably stable in bodily fluids, including blood; consequently, they are highly suitable for liquid‐biopsy applications. Because they are protected from enzymatic degradation by their lipid bilayers, microRNAs (miRNAs) within EVs have demonstrated significant potential for diagnosing various diseases; hence, they are reliable sources of biomarkers.^[^ ^4^ ^]^ In the context of atherosclerosis, several EV‐associated miRNAs, such as miR‐33a‐5p, miR‐126‐3p, and miR‐145‐5p, have been identified as biomarkers with significant diagnostic potential owing to their involvement in critical processes, including lipid accumulation, endothelial dysfunction, and plaque stabilization.^[^ ^5^ ^]^

While the highly sensitive and specific reverse transcription–quantitative polymerase chain reaction (RT‐qPCR) is most widely used to detect EV miRNAs, this technique requires extensive sample preparation, including EV lysis, which is labor‐intensive, time‐consuming, and prone to sample loss. Moreover, RT‐qPCR often requires expensive reagents and sophisticated equipment, which limits its applicability to routine clinical use. Alternative approaches, such as vesicle‐fusion‐based methods, have demonstrated promise by enabling the direct analysis of EV contents.^[^ ^6^ ^]^

**Scheme 1:**
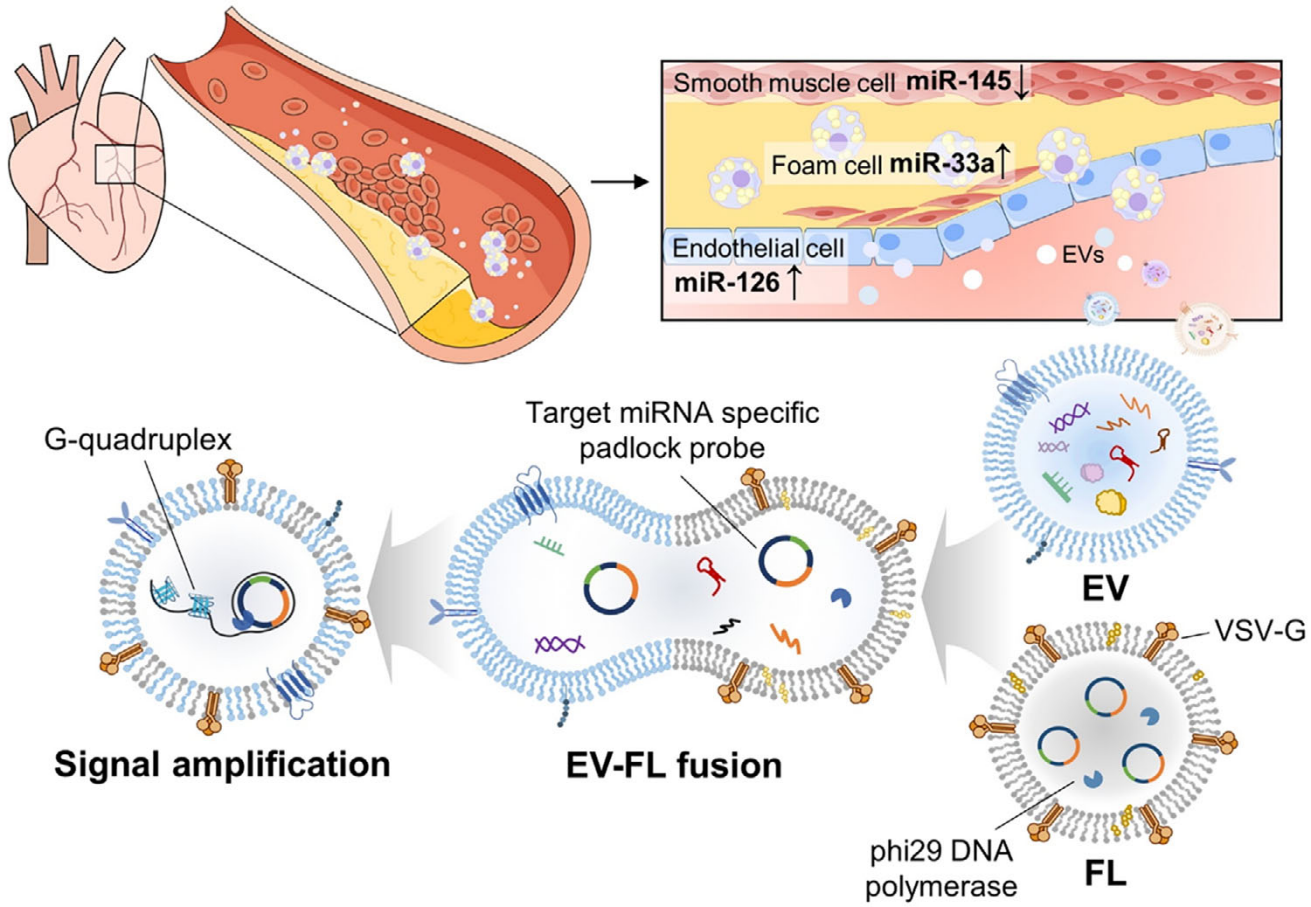
Fusogenic nanoreactor‐based EV‐miRNA detection method for diagnosing atherosclerosis. We developed a novel vesicle fusion‐based platform for direct detection of EV miRNAs, enabling highly specific and sensitive disease stage discrimination. By engineering FLs carrying VSV‐G and reagents for RCA, we achieved target‐specific signal generation within intact EVs without RNA extraction or reverse transcription. This platform detects three miRNAs (miR‐33a, miR‐126, and miR‐145) via RCA‐triggered G‐quadruplex formation, allowing fluorescence‐based quantification. The system was validated using cell‐derived, mouse, and human serum EVs, exhibiting robust diagnostic performance for atherosclerosis – an asymptomatic, artery‐narrowing disease with limited blood‐based diagnostic options. By capturing EVs released from plaque, our method offers a minimally invasive alternative. The method simplifies the workflow, reduces the sample loss and cost, and maintains high analytical accuracy through direct in situ detection of EV miRNAs.

In the present study, we developed a vesicle‐fusion‐based EV miRNA detection platform and demonstrated its ability to highly specifically and sensitively distinguish disease stages. Specifically, fusogenic liposomes (FLs) were designed to carry vesicular stomatitis virus glycoprotein (VSV‐G) on their surfaces, which facilitates fusion with EVs and encapsulates reagents for rolling circle amplification‐based (RCA‐based) signal amplification. RCA exhibits exceptional specificity owing to its requirement for target sequence recognition by a padlock probe, minimizing the risk of nonspecific amplification. By integrating RCA into our fusion‐based detection platform, we achieved highly sensitive and specific EV miRNA detection directly within intact EVs, maintaining high analytical accuracy. We constructed three distinct FLs, each tailored to detect one of three specific miRNAs, evaluated the performance of the platform for detecting target EV miRNAs using EVs derived from cells and mouse and human sera, and explored its ability to diagnose atherosclerosis (**Scheme** **1**). Following successful fusion between EVs and FLs, target‐specific RCA reactions were initiated within the EVs, leading to the generation of G‐quadruplex structures as the final reaction products. These structures enabled fluorescence‐based detection, allowing the quantification of EV miRNA expression levels with high sensitivity and specificity. Notably, the developed method eliminates unnecessary sample processing steps, specifically RNA extraction from EVs and reverse transcription required in RT‐qPCR, which often lead to sample loss, long analysis times, and high costs. Probe‐sequence modification ensures that the developed approach is readily adaptable to broader diagnostic applications by detecting a variety of miRNAs, thereby underscoring its versatility and potential clinical impact.

## 2 Results and Discussion

### 2.1 Fabricating and Characterizing FLs and EVs

**Figure 1:**
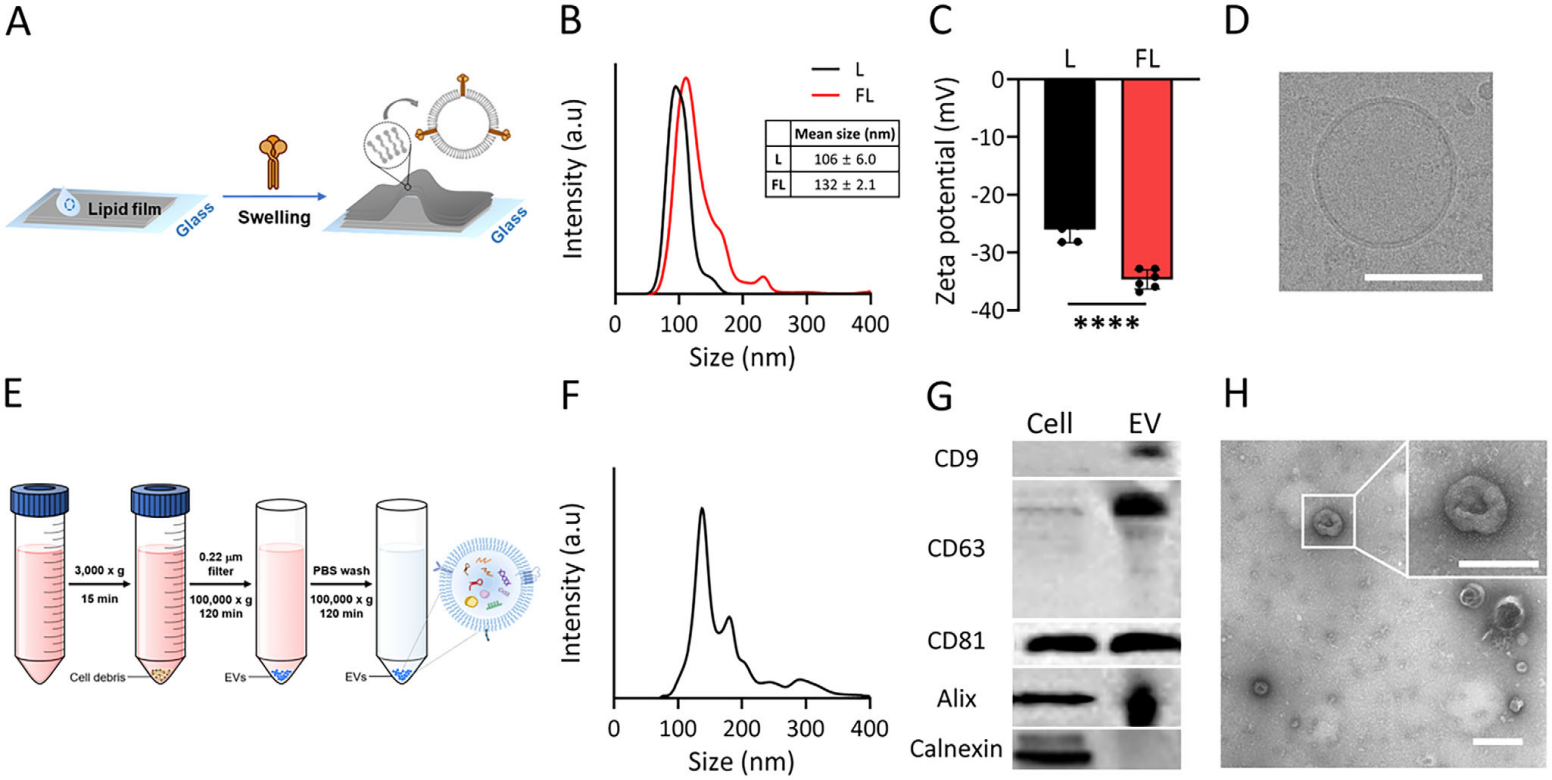
Characterizing FLs and HEK293T‐cell‐derived EVs. A) Schematic depicting FL production. B) Size‐distribution profiles for liposomes with (FL) and without VSV‐G (L). C) Zeta potentials of FLs; *n* = 5–6, Student's t‐test. D) Cryo‐TEM image of FLs. Scale bar: 100 nm. E) Schematic depicting EV production. F) Size‐distribution profile for EVs. G) Western‐blot analysis of EV marker proteins (CD9, CD63, CD81, Alix) and a non‐EV protein (calnexin). Cell lysate and EV samples were loaded for comparison. H) TEM image of EVs. Scale bar: 100 nm. All values are means ± SDs: ****p < 0.0001.

We first characterized the engineered FLs and EVs isolated from HEK293T cells to establish a vesicle‐fusion‐based EV miRNA detection platform. VSV‐G, which is known for its ability to mediate membrane fusion between vesicular stomatitis virus and host cells under low pH conditions, has been widely used as a fusogen in studies into drug delivery, macromolecular transfer, and genetic modification using cells, plasma membrane vesicles, and EVs.^[^ ^7^ ^]^ FLs were designed to display VSV‐G fusogen proteins on their external surfaces by leveraging these properties, thereby facilitating membrane fusion with EVs (**Figure** **1**A). VSV‐G was transfected into HEK293T cells and validated by SDS‐PAGE and western blot analysis (Figure S1, Supporting Information). The liposome size distribution profile reveals a monodispersed population with an average diameter of 106 nm; this value was higher (132 nm) after incorporating VSV‐G (Figure 1B). The FLs exhibited a significantly more negative zeta potential compared to that of the VSV‐G‐free liposomes, further confirming that VSV‐G had been successfully localized on the FL surfaces (Figure 1C). Additionally, cryo‐transmission electron microscopy (TEM) revealed that the FLs have a uniformly spherical morphology and are size‐consistent, in agreement with the size‐distribution data (Figure 1D).

EVs were isolated from the HEK293T‐cell culture medium by ultracentrifugation and characterized to confirm that they had been properly acquired (Figure 1E). Size distribution data revealed that the majority of the EV population was 100–200 nm in size (Figure 1F). Western blotting revealed the presence of EV‐specific markers (CD9, CD63, CD81, and Alix), whereas the absence of calnexin, a non‐EV marker, is indicative of minimal contamination by cellular debris (Figure 1G). The vesicular EV morphology was further confirmed by TEM, with sizes within the expected range (Figure 1H).

### 2.2 FL/EV Membrane Fusion by VSV‐G

**Figure 2:**
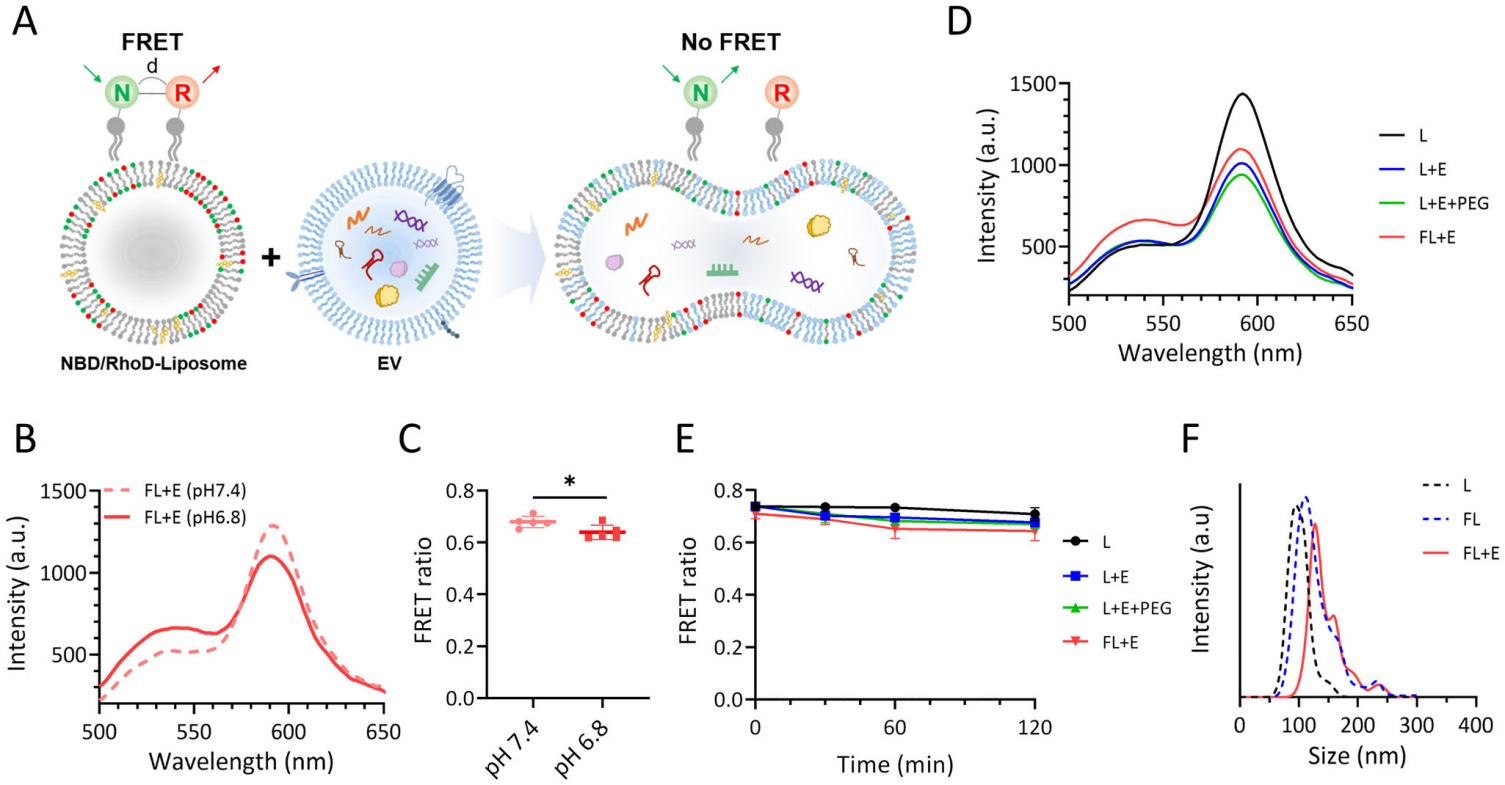
Evaluating FL and EV membrane fusion using a FRET‐based assay. A) Schematic depicting the FRET‐based detection of FL/EV membrane fusion. FLs are labeled with a donor–acceptor FRET pair, with fusion leading to a reduction in FRET. B) Emission spectra of the donor fluorophore and C) corresponding FRET ratios at various pH values; *n* = 5, Student's t‐test. D) FRET spectra after a 2‐h fusion reaction and E) corresponding FRET ratios that compare fusion efficiencies using different fusion methods; *n* = 3. F) Size‐distribution profiles of L, FLs, and FL‐EV mixtures after fusion reactions. All values are means ± SDs: *p < 0.05.

FLs and EVs need to be efficiently fused to directly detect EV miRNAs without additional sample processing. Membrane fusion was validated by labeling FLs with the nitrobenzoxadiazole (NBD, donor)/rhodamine B (Rhod, acceptor) Förster resonance energy transfer (FRET) fluorophore pair; these fluorophores become more spatially separated upon EV fusion, which reduces FRET efficiency (**Figure** **2**A). Because VSV‐G undergoes a fusion‐promoting conformational change under acidic conditions,^[^ ^8^ ^]^ we compared fusion efficiencies in physiological (pH 7.4) and slightly acidic (pH 6.8) environments. The fluorescence emission spectra and corresponding FRET ratios revealed significantly enhanced fusion at pH 6.8 compared to pH 7.4 (Figure 2B,C). We also assessed how the VSV‐G content affects membrane fusion. Increasing the VSV‐G content from 1 to 5 wt.% relative to the lipid film improved fusion stability and efficiency (Figure S2, Supporting Information) in a similar manner to that observed for the protein‐to‐lipid ratios of fusogens reported in previous studies.^[^ ^9^ ^]^

We compared the VSV‐G‐mediated fusion efficiency using the optimized FL formulation with those of alternative approaches, including non‐fusion liposomes (L+E) and the commonly used polyethylene‐glycol‐induced (PEG‐induced) fusion (L+E+PEG) agent.^[^ ^10^ ^]^ The FLs prepared using the method developed herein exhibited the highest fusion efficiency, as demonstrated by fluorescence emission spectroscopy and FRET ratio calculations (Figure 2D,E). The FL‐EV mixture exhibited larger particles in its size distribution profile following fusion (Figure 2F). Cryo‐TEM imaging was used to confirm that the larger particles are ascribable to membrane fusion rather than aggregation. Figure 2G shows that: a) the FLs and EVs are initially in close proximity, b) the membranes then fuse, and c) enlarged vesicles are formed, suggestive of successful vesicle fusion. These results demonstrate that the VSV‐G‐mediated fusion approach ensures that the vesicles interact efficiently; consequently, this approach is suitable for use in downstream biomarker detection applications.

### 2.3 Feasibility of Detecting RCA‐Based miRNA

**Figure 3:**
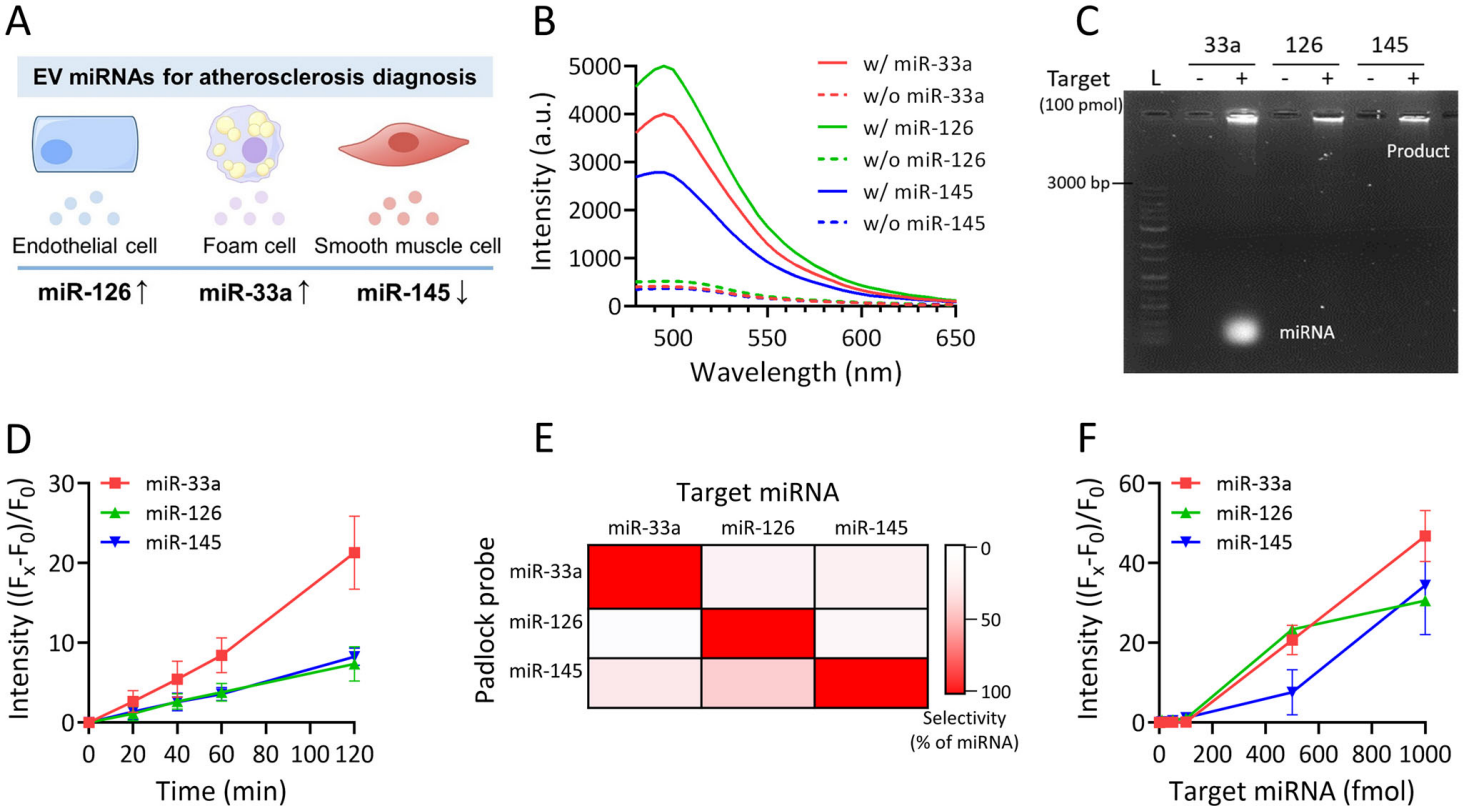
Sensitivity and selectivity of the RCA‐based miRNA detection method. A) Schematic depicting the three EV‐associated miRNA targets (miR‐33a‐5p, miR‐126‐3p, and miR‐145‐5p) selected for diagnosing atherosclerosis. B) Emission spectra and C) gel electropherograms of RCA reactions performed in the presence (w/) or absence (w/o) of the three target miRNAs. In (B), solid lines represent reactions containing the respective target miRNA, and dashed lines indicate reactions conducted without the target miRNA. D) Reaction‐time‐dependent RCA reaction intensities; *n* = 3. E) Selectivity of each padlock probe for its corresponding miRNA target. F) Target miRNA‐concentration‐dependent RCA reaction intensities; *n* = 3. All values are means ± SDs.

We focused on miR‐33a‐5p, miR‐126‐3p, and miR‐145‐5p as potential diagnostic targets when evaluating the potential of our platform for detecting EV miRNAs (**Figure** **3**A). These miRNAs reportedly exhibit significant concentration gradients that correlate with the coronary‐atherosclerosis phenotype and are recognized for their strong discriminating power for predicting the presence of the disease.^[^ ^5^ ^]^ The encapsulated padlock probe is expected to become involved in RCA reactions specifically initiated by EV miRNAs following FL‐EV fusion. The RCA reaction results in a significant increase in fluorescence only in the presence of the corresponding target miRNA when a target‐specific padlock probe is used (Figure 3B). This notion was further confirmed by gel electrophoresis, which revealed that only samples containing the target miRNAs exhibited high‐molecular‐weight RCA products (Figure 3C). Moreover, excluding nonspecific reactions from other components, the observed RCA products were specifically generated by the target miRNAs (Figure S3, Supporting Information). Fluorescence intensity was observed to increase linearly with reaction time under the optimized RCA conditions (Figure 3D; Figure S4, Supporting Information). Each padlock probe generated strongly detectable signals solely with its corresponding target miRNA, thereby demonstrating excellent target–probe specificity (Figure 3E). Furthermore, fluorescence intensity was found to correlate with the concentration of the target miRNA, even at low concentrations (0–100 fmol) (Figure 3F; Figure S5, Supporting Information). The platform exhibited a low limit of detection (LOD) of 50 fmol and was highly sensitive and accurate, with a linear detection range clearly observed (Figure S6, Supporting Information). Taken together, these results show that RCA reactions facilitated by the designed padlock probes enable target miRNAs to be effectively and sensitively detected.

### 2.4 In Situ EV‐miRNA Analysis via fRCA

**Figure 4:**
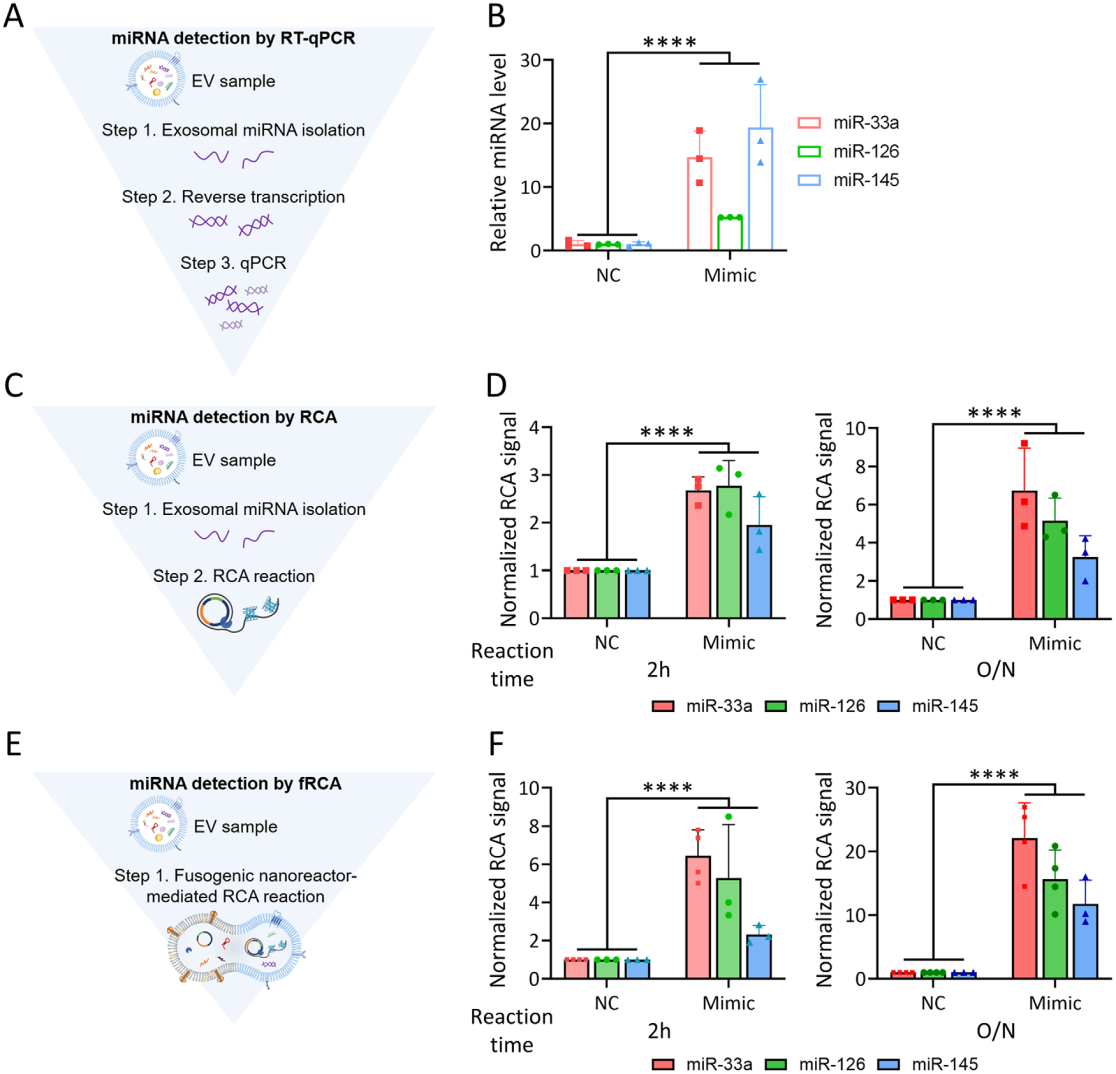
Feasibility of fRCA for analyzing EV miRNA in situ. A,C,E) Schematic depicting the analysis steps and B,D,F) normalized detection‐signal data for the three EV‐miRNA‐detection methods using: a commercially available PCR kit for RNA extraction and reverse transcription (A–B, RT‐qPCR), an RCA reaction performed after extracting the RNA from the EVs (C–D, RCA), and a membrane‐fusion‐based RCA reaction without RNA isolation (E–F, fRCA). Note that RCA reactions were conducted under two different time conditions: 2 h and overnight (O/N), *n* = 3–4. All values are means ± SDs: ****p < 0.0001.

After validating the RCA‐based design for effectively detecting miRNAs by confirming that the FLs and EVs had been successfully fused, we hypothesized that the fusion RCA (fRCA) combination would enable the efficient in situ detection of EV miRNAs. This approach was validated by transfecting HEK293T cells with miRNA mimics that overexpress the target miRNAs in EVs. We compared three EV‐miRNA analysis methods. Accordingly, the EVs were first lysed using a commercial RT‐qPCR kit followed by reverse transcription and qPCR analysis (**Figure** **4**A), which revealed that the transfected EVs have elevated miRNA levels compared to the negative control (NC) mimic control, which confirmed that miRNAs had been successfully overexpressed (Figure 4B). RCA reactions were then conducted either for 2 h or overnight (O/N) after lysing the EVs and extracting their RNA (Figure 4C). While both reaction times led to significant signals, those from the O/N reactions were more intense. Despite this, 2 h was found to be sufficient for detection, which demonstrates the efficiency of the RCA reaction (Figure 4D). The fRCA method avoids the need for EV lysis through FL/EV fusion and by directly encapsulating padlock probes within intact vesicles (Figure 4E). This approach was found to generate stronger signals than RCA with EV lysis, most likely because RNA‐extraction losses are eliminated. Significant signals were observed even using 2‐h fRCA reactions, with miR‐33a‐5p and miR‐126‐3p levels comparable to those obtained using RT‐qPCR (Figure 4F). Interestingly, miR‐145‐5p exhibited less‐intense signals when either RCA or fRCA was used compared to RT‐qPCR. This discrepancy arises from differences in the detection principles of the two methods. RT‐qPCR amplifies reverse‐transcribed RNA and depends on extraction efficiency, whereas fRCA directly detects miRNAs within intact EVs. Additionally, variations in reaction conditions, including buffer composition, probe structure, target binding sensitivity to the probe, and reaction efficiency, contribute to the differing trends observed between the two techniques. We assessed the encapsulation efficiency (EE) for each target miRNA with the aim of evaluating FL performance in the encapsulating padlock probes; EEs were found to range between 20% and 35% with an average value of ≈25% (Figure S7, Supporting Information). These values are consistent with those previously reported for similar systems.^[^ ^11^ ^]^ We adjusted the input amounts in the RCA reactions to ensure accurate quantification by considering the average EE values. Overall, these findings show that our fRCA method enables the rapid and efficient detection of EV miRNAs without the need for lysis, thereby significantly simplifying the workflow.

### 2.5 Validating the fRCA‐Mediated Detection of EV miRNAs for Diagnosing Atherosclerosis in Mice

**Figure 5:**
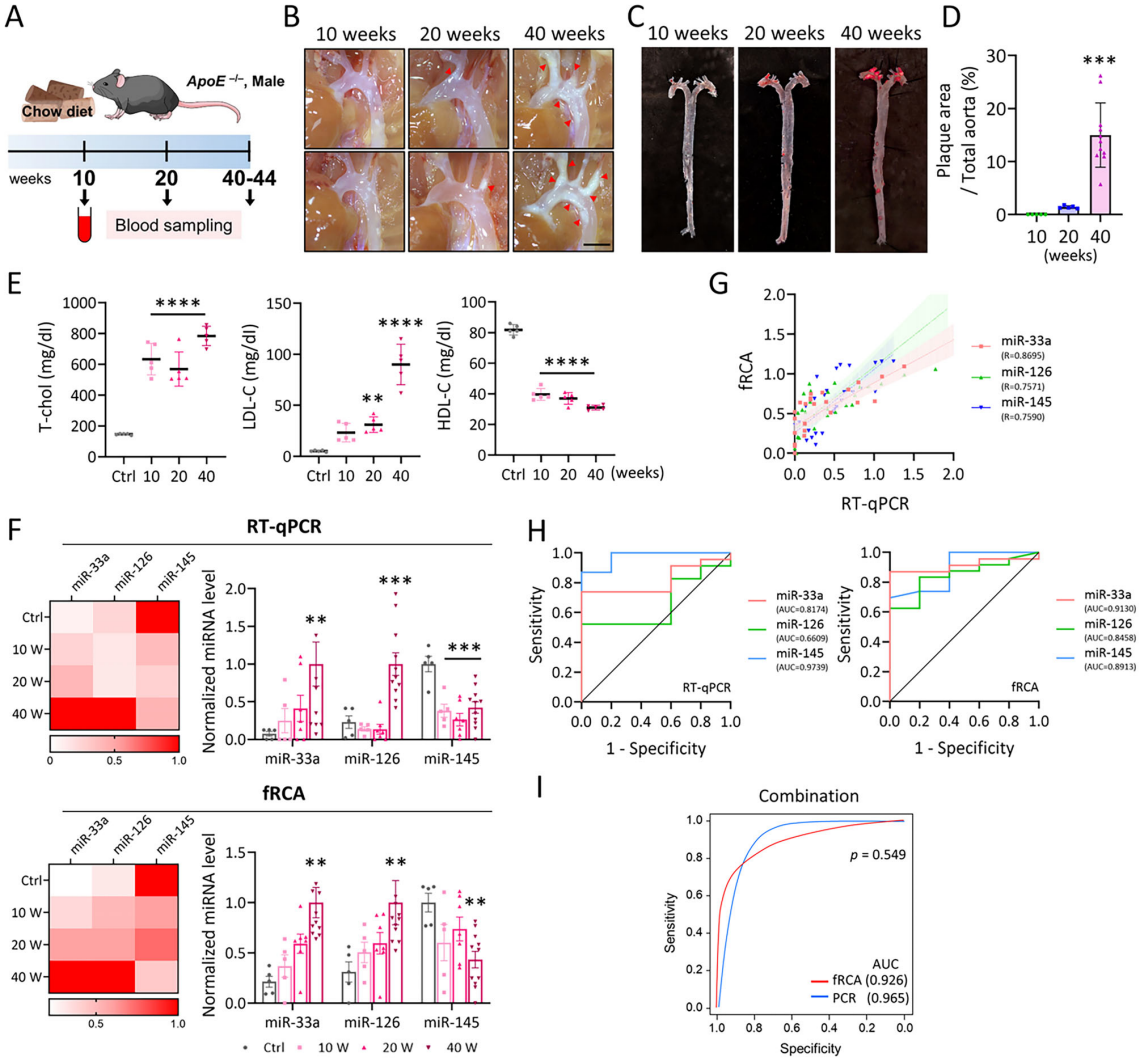
In vivo detection of EV miRNAs via fRCA during atherosclerosis diagnosis. A–E) Male *ApoE* ^−/−^ mice were fed a normal chow diet for 10, 20, or 40–44 weeks (*n* = 5, 5, and 11, respectively). A) Schematic depicting the feeding protocol and time points for serum isolation at each stage. B) Representative photographic images of atherosclerotic plaques on the aortic arch. Scale bars: 0.2 cm. Red triangles indicate plaque lesions at respective stages. C) Representative *en‐face* images of Oil‐Red‐O‐stained whole aortas at different time points. D) Quantifying plaque areas as percentages of the total aorta. Each data point represents an individual mouse; one‐way ANOVA. E) Serum levels of T‐chol, LDL‐C, and HDL‐C in normal C57BL/6J control (Ctrl) mice (*n* = 5; 10 weeks old) and *ApoE* ^−/−^ mice at 10, 20, and 40 weeks (*n* = 5 for each group). Each data point represents an individual mouse; one‐way ANOVA. F) Detecting EV miRNAs using the RT‐qPCR (top) and fRCA (bottom) methods. Each data point represents an individual mouse. G) RT‐qPCR/fRCA correlation analysis for miR‐33a, miR‐126 and miR‐145; Pearson correlation analysis. H) ROC curves and AUC analyses for miR‐33a, miR‐126, and miR‐145 RT‐qPCR data (left) and fRCA data (right). I) ROC curves and AUC analyses of the logistic regression model based on the combination of miR‐33a, miR‐126, and miR‐145, for RT‐qPCR data (blue) and fRCA data (red). All values are means ± SDs: **p < 0.01, ***p < 0.001, and ****p < 0.0001. Statistical significance was analyzed relative to the control group (Ctrl). T‐chol, total cholesterol; LDL‐C, low‐density lipoprotein cholesterol; HDL‐C, high‐density lipoprotein cholesterol; ANOVA, analysis of variance; W, weeks.

The fRCA‐mediated atherosclerosis diagnosis system was validated in mouse models by investigated the levels of EV miRNAs, total cholesterol (T‐chol), low‐density lipoprotein‐cholesterol (LDL‐C), high‐density lipoprotein‐cholesterol (HDL‐C), and atherosclerotic plaques during the various stages of atherosclerosis, including healthy, fatty streaks, intermediated lesions, and advanced lesions, in C57BL/6 and apolipoprotein‐E‐deficient (*ApoE^−/−^*) mice. The *ApoE^−/−^* mice were fed an NC diet, euthanized at 10, 20, or 40–44 weeks, and the progression of the atherosclerosis analyzed, with 10‐week‐old C57BL/6 mice used as healthy controls (**Figure** **5**A). Dual‐energy X‐ray absorptiometry (DEXA) was used to examine compositional changes in the bodies of the *ApoE^−/−^* mice at different ages, which revealed significant age‐dependent differences in body weight and lean mass (Figure S8A, Supporting Information). Although fat mass did not significantly change, the epididymal white adipose tissue (eWAT) to body weight ratio tended to increase with age; however, the weights of the livers of the *ApoE^−/−^* mice appeared to be independent of age. Atherosclerotic plaque areas were analyzed by isolating mouse aortas and examining plaque formation in the aortic arches and whole aortas. The aortas of 10‐week‐old C57BL/6 mice did not show atherosclerotic plaques (data not shown); however, the aortas of *ApoE^−/−^* mice developed age‐dependent atherosclerotic plaques, from fatty streaks to advanced lesions (Figure 5B–D). In addition, the sera of the *ApoE^−/−^* mice exhibited markedly higher levels of T‐chol and significantly lower LDL‐C and HDL‐C levels compared to those of the normal C57BL/6 mice (control, Ctrl) (Figure 5E).

First, to characterize EVs extracted from Ctrl and *ApoE^−/−^* mouse serum, we performed NTA, TEM, and protein marker analyses (CD63, CD81, and Alix), confirming successful EV isolation (Figure S9A,B, Supporting Information). According to these mouse serum‐derived EVs, we assessed the diagnostic performance of the fRCA. RT‐qPCR first revealed significantly higher expression levels of miR‐33a and miR‐126 in *ApoE^−/−^* mouse serum EVs, whereas miR‐145 levels were notably lower compared with the levels observed for the Ctrl mice. These findings were further corroborated by additional fRCA analyses, which consistently revealed higher miR‐33a and miR‐126 levels and lower miR‐145 levels in *ApoE^−/−^* mouse‐serum EVs relative to those of the control (Figure 5F). To verify target specificity, we performed an additional experiment to confirm that the signal observed in the nanoreactor was specifically generated by the padlock probe–mediated reaction. In the absence of the padlock probe (blank), the signal from mouse serum‐derived EVs remained negligible across all samples and stages, supporting the high specificity of our approach (Figure S9C, Supporting Information). Furthermore, correlation analysis revealed that the RT‐qPCR and fRCA data for the detection of miR‐33a (R = 0.8695), miR‐126 (R = 0.7571), and miR‐145 (R = 0.7590) are positively correlated (Figure 5G). These results suggest that fRCA is a diagnostic system comparable to RT‐qPCR.

Diagnostic performance was then validated using receiver operating characteristic (ROC) and area under the curve (AUC) analyses to determine whether or not the values obtained using RT‐qPCR and the fRCA method are capable of distinguishing between the control and atherosclerotic mice. RT‐qPCR was highly diagnostically accurate for miR‐33a (AUC: 0.8174), miR‐126 (AUC: 0.6609), and miR‐145 (AUC: 0.9739). Similarly, the fRCA method demonstrated comparable diagnostic accuracies for miR‐33a (AUC: 0.913), miR‐126 (AUC: 0.8458), and miR‐145 (AUC: 0.8913); these data validate the diagnostic performance of the developed method (Figure 5H). Additionally, logistic regression‐based combination analysis of the three miRNAs demonstrated high diagnostic accuracy, with AUC values of 0.965 for RT‐qPCR and 0.926 for fRCA (Figure 5I).

### 2.6 Validation of fRCA‐Based Detection of EV miRNAs for Diagnosing Individuals at High‐Risk of Atherosclerosis

**Figure 6:**
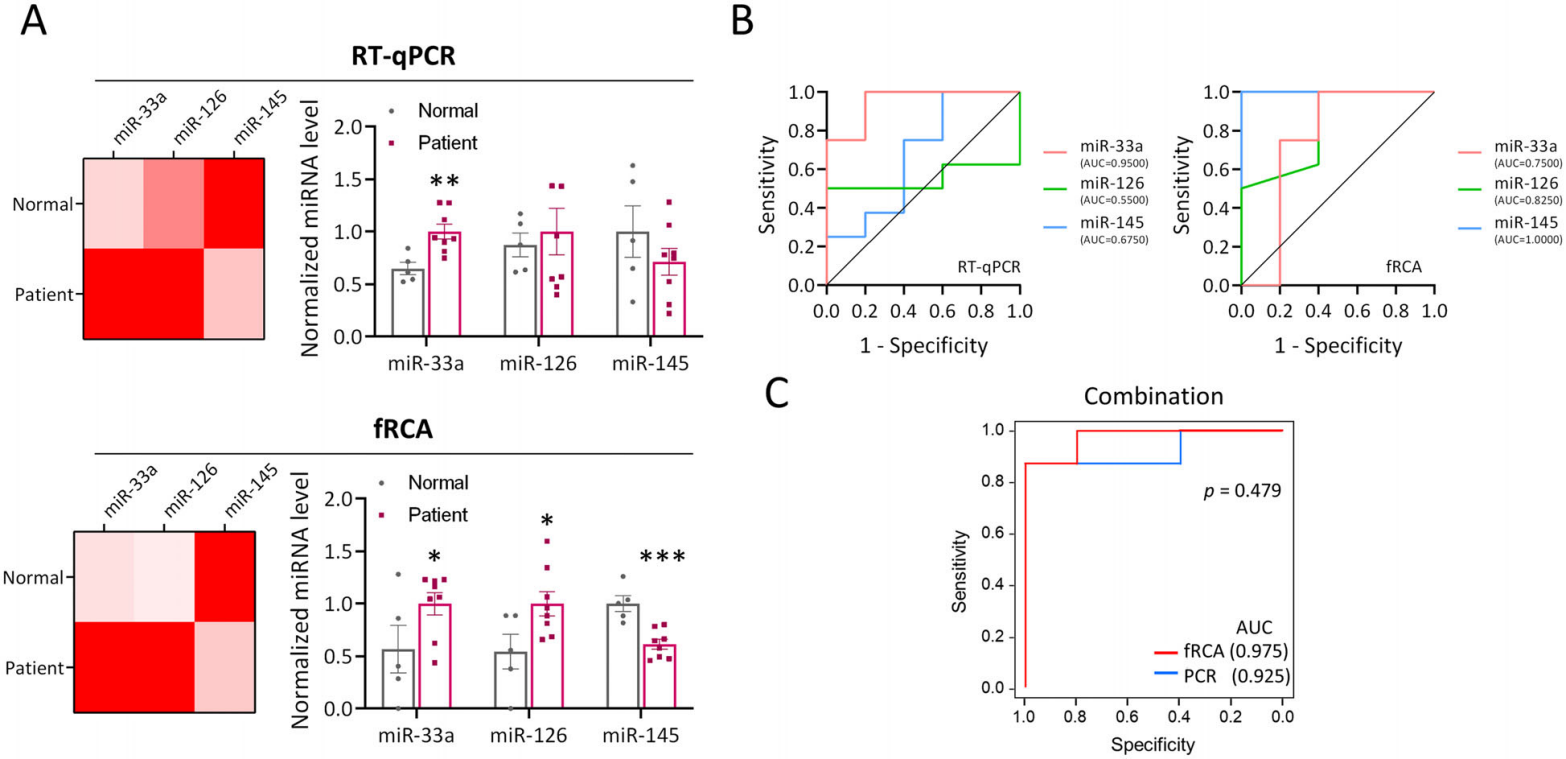
Detecting EV miRNAs via fRCA during atherosclerosis diagnosis in humans. A) Detecting EV miRNAs using the RT‐qPCR (top) and fRCA (bottom) methods. Each data point represents an individual. B) ROC curves and AUC analyses for miR‐33a, miR‐126, and miR‐145 RT‐qPCR data (left) and fRCA data (right). C) ROC curves and AUC analyses of the logistic regression model based on the combination of miR‐33a, miR‐126, and miR‐145, for RT‐qPCR data (blue) and fRCA data (red). All values are means ± SDs: **p < 0.01, ***p < 0.001, and ****p < 0.0001. Statistical significance was analyzed relative to the normal group. Normal, healthy individual; Patient, patient with coronary artery disease.

According to the validation of fRCA performance in mouse models, we further aimed to evaluate the applicability of the fRCA system to human serum‐derived EVs (Figure S9A,B, Supporting Information). To this end, we analyzed serum‐derived EV expression levels of miR‐33a, miR‐126, and miR‐145 using both the RT‐qPCR and fRCA methods in two groups: healthy individuals (Normal, *n* = 5), patients with coronary artery disease (Patient, *n* = 8). Although the patient cohort was small, the results showed a trend of increased expression levels of miR‐33a and miR‐126 in the patient groups compared to healthy controls, while miR‐145 expression showed a decreasing trend in the patient groups (**Figure** **6**A). These expression patterns were consistent with the trends observed in mouse models, supporting the relevance of the findings.

In the ROC and AUC analyses, RT‐qPCR exhibited the highest diagnostic accuracy for miR‐33a, with an AUC of 0.95, while miR‐126 and miR‐145 had AUC values of 0.55 and 0.68, respectively. In contrast, fRCA demonstrated AUC values of 0.75 for miR‐33a, 0.83 for miR‐126, and 1.00 for miR‐145 (Figure 6B). When the three miRNAs were analyzed using a logistic regression‐based combination approach, both RT‐qPCR and fRCA exhibited high diagnostic accuracy, with AUC values exceeding 0.9 (Figure 6C).

In summary, the fRCA method, which was initially developed in vitro, exhibited diagnostic performance similar to that of RT‐qPCR when directly analyzing EVs from mouse and human blood samples. Given that the procedure is rapid, simple, and does not require temperature control, fRCA is anticipated to be more practical and efficient for clinical diagnostics than conventional PCR methods.

## 3 Conclusion

In this study, we introduced an all‐in‐one diagnostic system as a novel diagnostic platform capable of amplifying target genes through reactions between serum EVs and FLs. This system integrates padlock probes for amplification and enzymes within liposomes while exposing fusogenic proteins to the liposome surface, thereby enabling membrane fusion with the EVs. This system is designed to amplify specific miRNAs upon fusion. We developed padlock probes that specifically targeted miR‐33a, miR‐126, and miR‐145, which had previously been identified as diagnostic markers for atherosclerosis, and successfully validated the fRCA method both in vitro and in vivo. Notably, positive correlations were observed between the diagnostic performance of the fRCA method and RT‐qPCR, which highlights its reliability as a diagnostic tool. Furthermore, ROC analysis demonstrated that the fRCA system can effectively and accurately distinguish not only between normal and atherosclerotic mice but also between healthy individuals and those with a high risk of atherosclerosis in humans. In addition, a comparison of total detection times indicated that our fRCA method (2.5–3 h) significantly reduces the detection time compared with RT‐qPCR (4–4.5 h), achieving approximately 1.5‐fold faster detection. This time efficiency, combined with its accuracy and simplified workflow, underscores the potential of fRCA as a diagnostic tool. To the best of our knowledge, this is the first study to demonstrate the potential use of an fRCA‐based amplification method to diagnose atherosclerosis.

## 4 Experimental Section

### Materials

All oligonucleotides used in this study were synthesized and BioRP‐purified by Bioneer (Korea), and used without further processing, as listed in Table S1 (Supporting Information). Phospholipids, including 1,2‐dioleoyl‐sn‐glycero‐3‐phosphocholine (DOPC), 1,2‐dioleoyl‐sn‐glycero‐3‐phospho‐l‐serine (DOPS), 1,2‐dioleoyl‐sn‐glycero‐3‐phosphoethanolamine (DOPE), cholesterol (chol), 1,2‐dioleoyl‐sn‐glycero‐3‐phosphoethanolamine‐N‐(7‐nitro‐2‐1,3‐benzoxadiazol‐4‐yl) (ammonium salt) (18:1 NBD‐PE), and 1,2‐dioleoyl‐sn‐glycero‐3‐phosphoethanolamine‐N‐(lissamine rhodamine B sulfonyl) (ammonium salt) (18:1 Liss Rhod‐PE) were purchased from Avanti Polar Lipids (AL, USA).

### Preparing the Circular Padlock Probes

Three padlock probes specific for the target miRNA (miR‐33a‐5p, miR‐126‐3p, and miR‐145‐5p) were designed to initiate the RCA reaction. Each probe consisted of three regions: a binding site for the splint primer, one for the target miRNA, and a G‐quadruplex‐forming site. Equal amounts of template and splint DNA (5 µm) were incubated with T4 DNA ligase (Cat. #6023; Takara Bio Inc., Japan) at 16 °C for at least 3 h. The reaction mixture was loaded onto 4% agarose gel prepared with 1 × Tris‐acetate‐EDTA (TAE) buffer (LPS Solution, Korea). Electrophoresis was conducted for 2 h at 25 V, and the desired bands were extracted using a QIAquick Gel Extraction Kit (QIAGEN, Germany). The circular templates were validated using 1% agarose gel electrophoresis following the RCA reaction and stored at −20 °C until further use.

### Padlock‐Probe Sensitivity and Selectivity Testing

The prepared circular padlock probes (10 ng) were incubated with varying concentrations of target miRNAs (0, 1, 5, 10, 50, and 100 nm) in buffer (final volume: 20 µL). The padlock probes were selectivity tested in the presence or absence of the target miRNA. Reactions were conducted at 37 °C for 2 h, after which an aliquot of the sample (10 µL) was mixed with ThT solution (90 µL 10 µm) and the fluorescence emission was recorded using a Biotek Synergy H1 Multimode reader (Winooski, VT, USA) with excitation at 440 nm.

### Preparing the FLs

The FLs prepared in this study are lipid vesicles that encapsulate the RCA reaction materials within their inner compartments and present VSV‐G proteins on their surfaces. A thin lipid film was formed by evaporating a suspension containing lipids (0.1 mg) in chloroform (DOPC/DOPE/DOPS/chol = 5:2:2:1 mol%) under reduced pressure. The resulting lipid film was hydrated with the required padlock probe (100 ng), appropriate concentrations of dNTPs (Thermo Fisher Scientific), and phi29 DNA polymerase (New England Biolabs) in NaCl buffer. The lipid suspension was sonicated at amplitude 20 for 10 s in an ice bucket, followed by mixing with the VSV‐G proteins (10 µg). Monodispersed lipid vesicles were obtained by extruding the solution 40 times through a membrane with 100 nm pores using a mini‐extruder (Avanti Polar Lipids, AL, USA). The lipid vesicle solution was finally mixed with an equal volume of 0.1 m PB buffer (pH 6.8) and subjected to centrifugal ultrafiltration using Amicon (10 kDa MWCO) to remove excess substances and yield purified lipid vesicles containing VSV‐G and RCA materials (referred to as “FLs”).

### Cell Culturing and EV Isolation

HEK293T cells were cultured in Dulbecco's Modified Eagle's medium (DMEM; Welgene, Korea) supplemented with 10% (v/v) fetal bovine serum (FBS; Welgene, Korea) and 1% antibiotic‐antimycotic solution (Cytiva, MA, USA) in a humidified environment at 37 °C under 5% CO_2_. EVs with the three target miRNAs overexpressed were isolated by transfecting the cells with 5 nm of the required miRNA or a negative control mimic (Bioneer, Korea) using the lipofectamine RNAiMAX transfection reagent (Thermo Fischer Scientific) according to the manufacturer's instructions. The medium was replaced with fresh medium containing 10% (v/v) EV‐depleted FBS 16 h post‐transfection, and the cells were cultured for 48 h. EV‐depleted FBS was prepared by ultracentrifugation at 31200 rpm for 2 h using an Optima L‐100K ultracentrifuge (Beckman‐Coulter, CA, USA) equipped with a SW32 Ti rotor (Beckman‐Coulter) and stored at −80 °C until use. The culture medium was collected, centrifuged at 3000 × g for 15 min and filtered through a 0.22‐µm syringe filter (Advantec, Japan). EVs were isolated by ultracentrifugation as described above. The resulting EV pellet was washed with Dulbecco's Phosphate Buffered Saline (DPBS) in a second ultracentrifugation step and resuspended in PBS. Serum‐derived EVs were isolated using an ExoQuick Exosome Isolation and RNA Purification Kit (Cat. #EQ806A‐1; System Biosciences, CA, USA) according to the manufacturer's instructions. The mixture was centrifuged at 1500 × g for 30 min and the resulting EV‐containing pellet was resuspended in the same volume of nuclease‐free PBS as originally used. All centrifugation steps were carried out at 4 °C. EV characterization was performed in accordance with the Minimal Information for Studies of Extracellular Vesicles (MISEV2023) guidelines.^[^ ^12^ ^]^

### NTA and Zeta‐Potential Analysis

FL and EV size distributions were analyzed using nanoparticle tracking analysis (NTA) on a Nanosight NS300 system (Malvern, UK) with the camera level set to 14 and a detection threshold of five used. The zeta potentials of the FLs in the presence and absence of the VSV‐G protein was measured using a Zetasizer Nano ZS instrument (Malvern, UK) at 25 °C. Each sample solution was diluted with DPBS and measured in triplicate.

### TEM and cryo‐TEM Imaging

TEM images were acquired on a CM20 transmission electron microscope (Philips, Netherlands) at an accelerator voltage of 200 kV. Each sample was applied to a 400‐mesh copper grid with a Formvar/carbon coating (Ted Pella, USA). The samples was negatively stained with 2% (w/v) uranyl acetate and then dried at 25 °C prior to TEM imaging. Cryo‐TEM images of the FLs, EVs, or fusion complexes (equivolume mixtures of FLs and EVs) were acquired using a Glacios instrument (Thermo Fisher Scientific, MA, USA) at an accelerator voltage of 200 kV. Data were collected using EPU software (Thermo Fisher Scientific Inc.).

### Western Blot Analyses

EVs were dissolved in RIPA buffer (Thermo Fisher Scientific, MA, USA) and heated to 95 °C for 5 min. Proteins were separated using 10% SDS‐PAGE and transferred onto polyvinylidene oxide (PVDF) membranes. The membranes were blocked for 1 h with 3% (w/v) bovine serum albumin or 5% (w/v) skim milk in PBS containing 0.1% Tween‐20 (PBS‐T), after which they were incubated overnight at 4 °C with the following primary antibodies: CD9 (sc‐13118; Santa Cruz Biotechnology), CD63 (sc‐5275; Santa Cruz Biotechnology), CD81 (sc‐166029; Santa Cruz Biotechnology), Alix (2171; Cell Signaling Technology), calnexin (ab133615; Abcam), and VSVG (NB100‐2485; Novus Biologicals). The membranes were subsequently washed with PBS‐T and incubated at 25 °C for 1 h with anti‐mouse IgG horseradish‐peroxidase‐linked antibody (7076, Cell Signaling Technology, MA, USA) and anti‐rabbit IgG horseradish‐peroxidase‐linked antibody (7074, Cell Signaling Technology). Protein bands were visualized using an enhanced chemiluminescence (ECL) detection reagent (Cytiva, Germany) and images were captured using an iBright CL1500 instrument (Invitrogen, CA, USA).

### Preparing the VSV‐G Protein

The VSV‐G fragment was obtained from the pMD2.G plasmid (Cat. #12 259; Addgene) and subcloned into the pcDNA3.4‐TOPO vector (Thermo Fisher Scientific) to construct a recombinant mammalian expression vector. The vector was transfected into HEK293T cells, and the medium was replaced with fresh medium containing 10% (v/v) fetal bovine serum (FBS) after 16 h. The cells were cultured for an additional 48 h. Proteins were extracted from the cell lysate and dialyzed against PBS for 24 h. Expression levels were evaluated using SDS‐PAGE as previously described.

### Assessing Membrane Fusion via FRET

Fluorescently labeled FLs were prepared using NBD‐PE and Rhod‐PE, as described above. FLs were mixed with HEK293T EVs (10⁶ particles µL⁻¹) and incubated for 2 h at 37 °C. The effect of pH was investigated by preparing FL solutions at pH 6.8 or 7.4. Additionally, the efficiencies of the various fusion methods were evaluated using liposomes lacking VSV‐G or by replacing liposomes with a 5% (v/v) PEG8000 solution. FRET was determined by monitoring the fluorescence intensity of the donor (NBD‐PE) at 538 nm, with excitation at 467 nm using a Biotek Synergy H1 Multimode Reader (Winooski, VT, USA).

The FRET ratio was calculated using the equation:

(1) $$\mathrm{FRET}\;\mathrm{ratio}\;=\frac{I_{A}}{I_{A}+I_{D}}$$

where *I_A_* and *I_D_* are the fluorescence intensities of the acceptor (Rhod at 592 nm) and donor (NBD at 538 nm), respectively. The NBD recovery rate was calculated using the following equation:

(2) $$\mathrm{NBD}\;\mathrm{intensity}\;\mathrm{increase}\;\;\left(\%\right)=\frac{I\;-\;I_{0}}{I_{NBD}\;-\;I_{0}}\;\times 100$$

where *I* and *I_0_* are the NBD fluorescence intensities at 120 and 0 min, respectively, and *I_NBD_* is the intensity of the sample containing only NBD.

### Real‐Time Polymerase Chain Reactions

RNA was extracted from aliquots of samples (20–50 µL) using the miRNeasy Micro Kit (Qiagen, Germany) following the manufacturer's instructions. RNA concentration and purity were assessed using a Colibri Microvolume spectrophotometer (Titertek‐Berthold, Germany). Reverse transcription and RT‐PCR were performed using an HB miR Multi Assay Kit (HeimBiotek Inc., Korea) according to the manufacturer's protocol. cDNA was synthesized from RNA (50 ng) and RT‐PCR was conducted using a StepOnePlus Real‐Time PCR system (Applied Biosystems, CA, USA).

### Detecting EV miRNAs Using FLs

An FL solution specific to each target miRNA (10 µL) was incubated with the EV solution (10 µL) for 2 h at 37 °C. Mouse‐serum‐derived EV solutions were prepared by mixing each EV solution (6.5 µL) with DPBS (3.5 µL). Equal volumes of 20% Triton X‐100 was added to each sample, which was then incubated at room temperature for 5 min. The sample was diluted ten‐fold, and an aliquot of the diluted sample (20 µL) was mixed with ThT solution (10 mm). FL encapsulation efficiency was determined by measuring the concentration of the padlock probe using a Colibri Microvolume spectrophotometer (Titertek‐Berthold, Germany), with fluorescence emissions recorded as described previously.

### Mouse Model of Atherosclerosis

All animal experiments were approved by the Institutional Animal Care and Use Committee of the Korea Research Institute of Bioscience and Biotechnology (KRIBB‐AEC‐23071). *ApoE^−/−^ mice* (002052) were obtained from The Jackson Laboratory (Bar Harbor, Maine, USA), and genotyping was confirmed by PCR with primers and conditions provided on The Jackson Laboratory website. Experiments were performed using male *ApoE^−/−^* mice from F6–F8 generations. Mice were fed an NC diet containing 13.2% fat and 24.6% protein for 10, 20, or 40−44 weeks to progress atherosclerosis to varying degrees.

### Human Samples

The protocol for collecting human samples was approved by the Institutional Review Board of Severance Hospital, Seoul, Korea (serial number of IRB; 4‐2013‐0688). Written informed consent was provided by all participants before enrollment. Serum samples were obtained from healthy individuals (Normal, *n* = 5) and patients with coronary artery disease before statin treatment (Patient, *n* = 8).

### Sample Collection and Analysis

Blood samples from the orbital venous plexus were collected in capillary tubes (without sodium heparin). The collected blood was allowed to clot at room temperature for 15 min and centrifuged at 3000 rpm for 15 min in a refrigerated centrifuge to isolate the serum. The entire aorta, from the proximal ascending aorta to the bifurcation of the iliac artery, was carefully dissected and adventitial fat removed. Aortas were split longitudinally for *en‐face* analysis, pinned onto flat black silicone plates, and fixed overnight in 10% (v/v) phosphate‐buffered formalin. Fixed aortas were stained with Oil Red O for 4 h. After briefly washing with PBS, the stained aortas were digitally photographed at a fixed magnification. Total aortic and lesion areas were calculated using AxioVision software (Carl Zeiss, Jena, Germany). DEXA (iNSiGHT VET DXA, OsteoSys, Korea) was used to determine the body composition, including fat and lean mass, of each ApoE^−/−^ mouse. Additionally, the livers and epididymal white adipose tissue (eWAT) were carefully dissected, photographed, and weighed to determine weight changes at various time points during the progression of atherosclerosis.

### Diagnostic Performance of Multi‐Marker

To evaluate the diagnostic performance of the three combined miRNAs (miR‐33a, miR‐126, and miR‐145), a logistic regression model with Least Absolute Shrinkage and Selection Operator (LASSO) regularization was constructed using the glmnet() function in R. Prior to model construction, class imbalance was addressed by adjusting the sample sizes to ensure a balanced distribution between groups. The dataset was then randomly split into training and test sets at a 3:7 ratio. During model training, ten‐fold cross‐validation was performed to determine the optimal regularization parameter, ensuring robust model performance.

### Statistical Analysis

Experimental data were analyzed using GraphPad Prism 10.4 (GraphPad Software, CA, USA), with significance determined using the unpaired two‐tailed Student's t‐test, one‐way, or two‐way ANOVA. Pearson correlation analysis was performed to evaluate the correlation between RT‐qPCR and fRCA results. Data were presented as means ± SDs in all bar graphs. Statistical significance was set at *p* < 0.05. All values were measured in at least three independent experiments.

## Conflict of Interest

The authors declare no conflict of interest.

## Author Contributions

J.L., K.K., M.J.C., and T.S. contributed equally to this work. J.L., K.K., M.J.C., and T.S. performed the experiments and collected the data. J.L., T.S., Y.R., S.L., D.‐S.K., M.‐S.L., H.S.B., E.‐K.L., S.‐H.L., and G.T.O. analyzed and interpreted the data. J.L., K.K., M.J.C., S.L., J.‐S.K., J.‐G.P., and T.‐S.H. drafted and revised the manuscript. J.‐G.P. and T.‐S.H. confirm the authenticity of all the raw data. All authors have read and approved the final manuscript.

## Supporting information



Supporting Information

## Data Availability

The data that support the findings of this study are available from the corresponding author upon reasonable request.
