# Supplementary material for: Fusogenic Nanoreactor‐Based Detection of Extracellular Vesicle‐derived miRNAs for Diagnosing Atherosclerosis
Source: Small. 2025 Apr 21;21(23):2501789. doi: 10.1002/smll.202501789 (PMC12160682; doi:10.1002/smll.202501789)
Supplement: Supplementary file 1 — Supporting Information [file SMLL-21-2501789-s001.docx]

Supporting Information

Fusogenic Nanoreactor-based Detection of Extracellular Vesicle-derived miRNAs for Diagnosing Atherosclerosis

Jiyoon Lee, Kiyoon Kwon, Min Ji Cho, Taesang Son, Yuna Roh, Sugi Lee, Dae-Soo Kim, Moo-Seung Lee, Hyun Seung Ban, Jang-Seong Kim, Eun-Kyung Lim, Sang-Hak Lee, Goo Taeg Oh, Jong-Gil Park* and Tae-Su Han*

**Table S1**. Oligonucleotide sequences used in this study.

| Name | Sequence (5’→3’)^*^ |
| --- | --- |
| miR-33a-5p | GUG CAU UGU AGU UGC AUU GCA |
| miR-126-3p | UCG UAC CGU GAG UAA UAA UGC G |
| miR-145-5p | GUC CAG UUU UCC CAG GAA UCC CU |
| Splint | ATA GAC GTG ATT ATT GTG ATT TTA |
| Template_33a | Phosphate-AAT CAC GTC TAT TGC AAT GCA ACT ACA ATG CAC TAT CCC ACT TTT CCC TCG TCG ACC CTA ACC CTA AAA TCA CAA T |
| Template_126 | Phosphate-AAT CAC GTC TAT CGC ATT ATT ACT CAC GGT ACG ATA TCC CAC TTT TCC CTC GTC GAC CCT AAC CCT AAA ATC ACA AT |
| Template_145 | Phosphate-AAT CAC GTC TAT AGG GAT TCC TGG GAA AAC TGG ACT ATC CCA CTT TTC CCT CGT CGA CCC TAA CCC TAA AAT CAC AAT |
| ^*^ The colors of oligonucleotide sequences correspond to those of the domains depicted in Scheme 1.  Green and red colors represent the sequence complementary to target miRNA and the G-quadruplex forming sequence, respectively. Underlined letters in Template represent the sequence complementary to splint DNA. | |


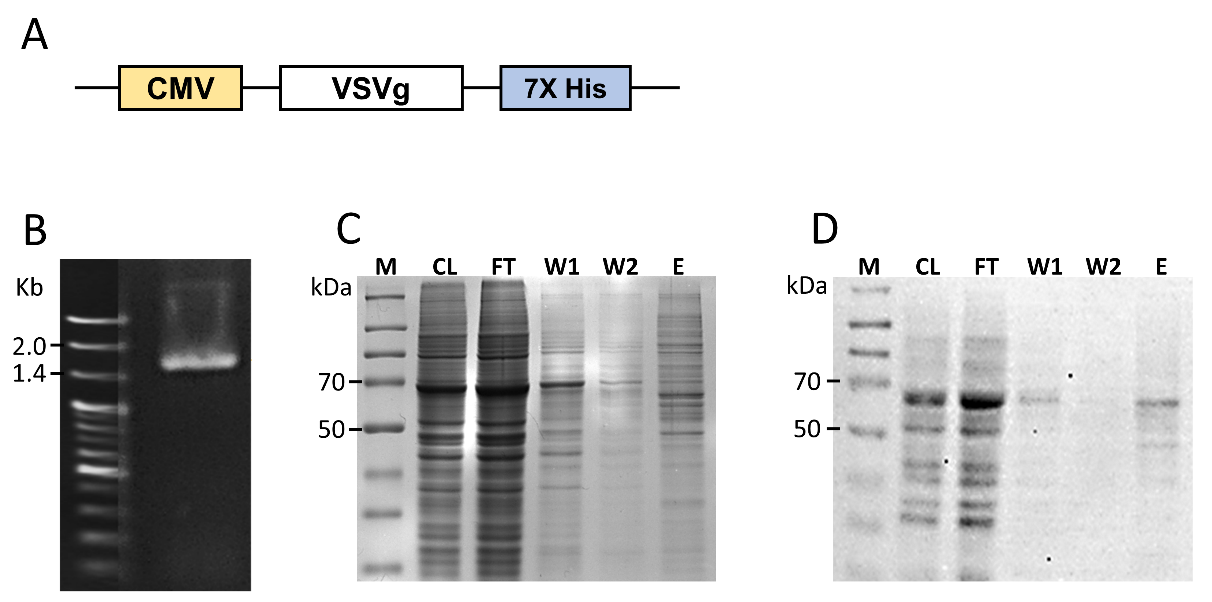


**Figure S1.** Purifying a VSV-G fusogen protein. (A) Schematic depicting the plasmid vector designed for expressing VSV-G. (B) Agarose gel electrophoresis that confirms the size of the extracted plasmid. (C) SDS-PAGE and (D) western-blot analyses of the purified VSV-G protein following His-tag-affinity chromatography (M: protein ladder, CL: cell lysate, FT: flow through, W: wash, E: eluate).


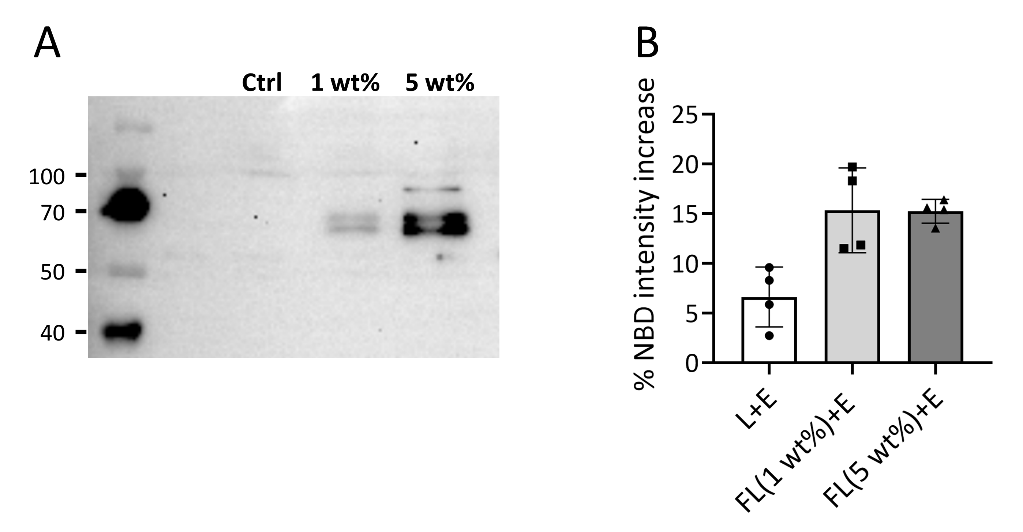


**Figure S2.** Determining the FL VSV-G protein ratio. (A) Western blot analysis of purified liposomes at various protein contents. (B) Quantifying fusion efficiency based on the percentage increase in NBD fluorescence after incubating three different liposome samples with EVs.


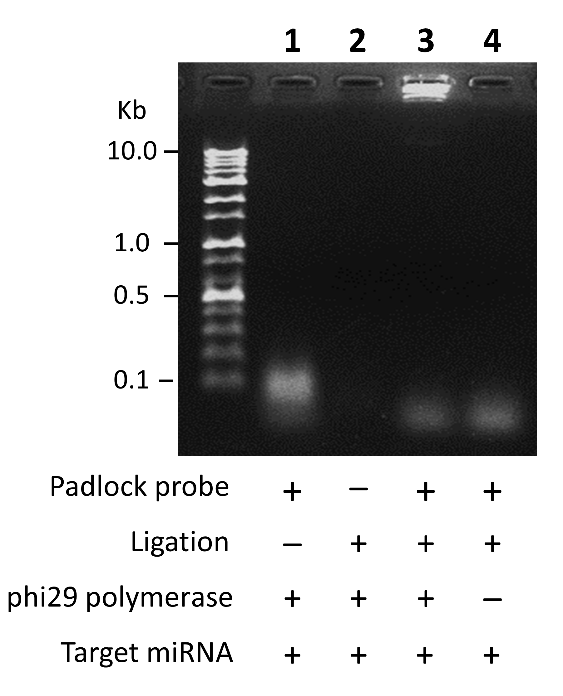


**Figure S3.** Characterizing RCA reactions performed under different conditions. Agarose-gel electropherogram of RCA reaction samples acquired under various reaction conditions. The first lane shows a 1 kb DNA ladder, with miR-33a and its corresponding padlock probe included under the tested conditions.


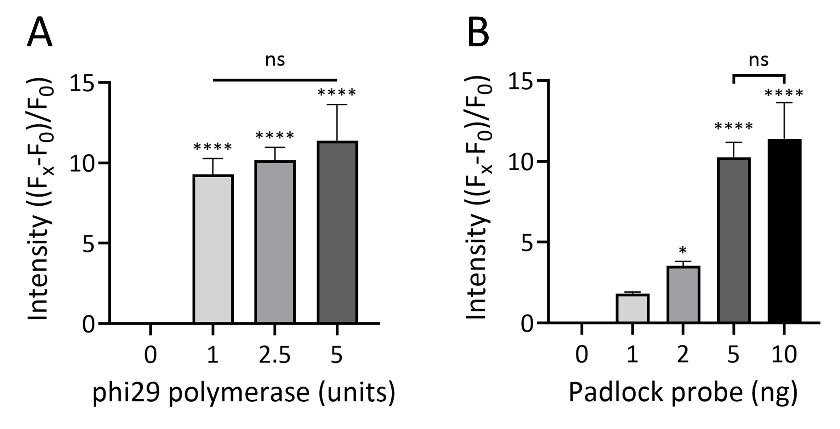


**Figure S4.** Optimizing the RCA reaction conditions by examining various concentrations of (A) phi29 DNA polymerase and (B) padlock probe, using 10 pmol of the miR-33a target. *n* = 3; **p* <0.03, *****p* < 0.0001, ns: not significant.


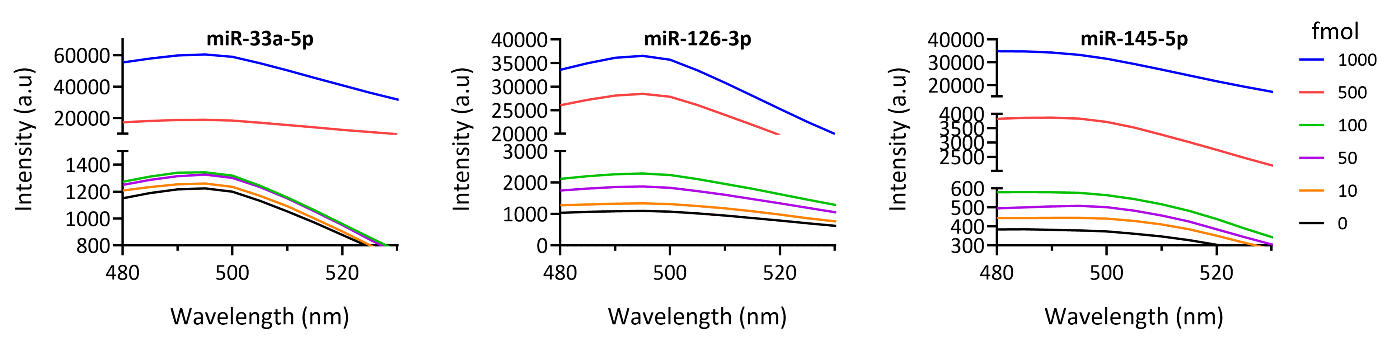


**Figure S5.** Fluorescence-emission spectra of RCA products at various target miRNA concentrations. The padlock probe corresponding to each target was used. The reaction was performed for 2 h, and an excitation wavelength of 440 nm was used.


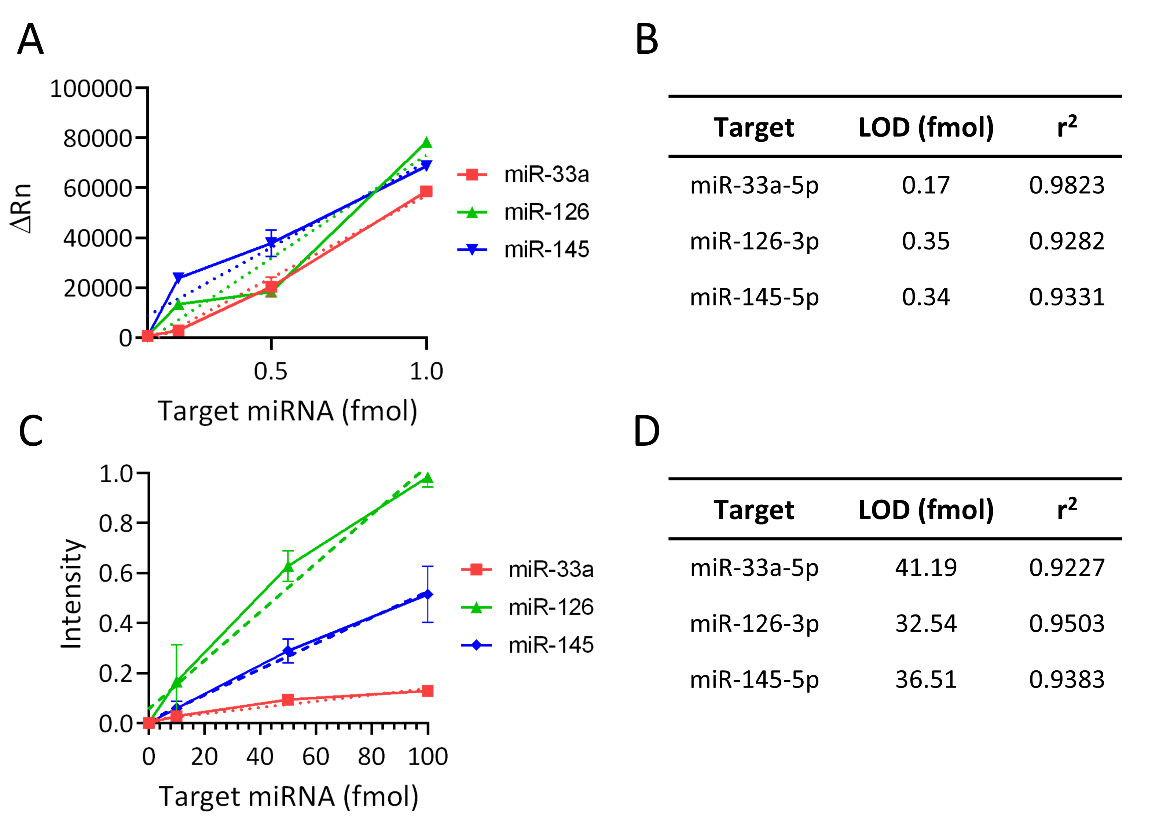


**Figure S6.** Determining the detection limits of the RT-qPCR and RCA methods. Signal measurements and limits of detection (LOD) for the three target miRNAs were evaluated at various concentrations using (A, B) RT-qPCR and (C, D) RCA. LODs were calculated as 3SD/slope, where SD is the standard deviation of the blank sample, and the slope is derived from the calibration curve.


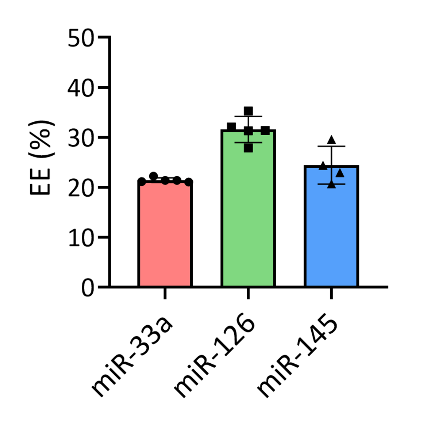


**Figure S7.** EEs (%) of the FL padlock probes calculated as the ratio of each padlock probe concentration encapsulated by the FLs to the initial input concentration.


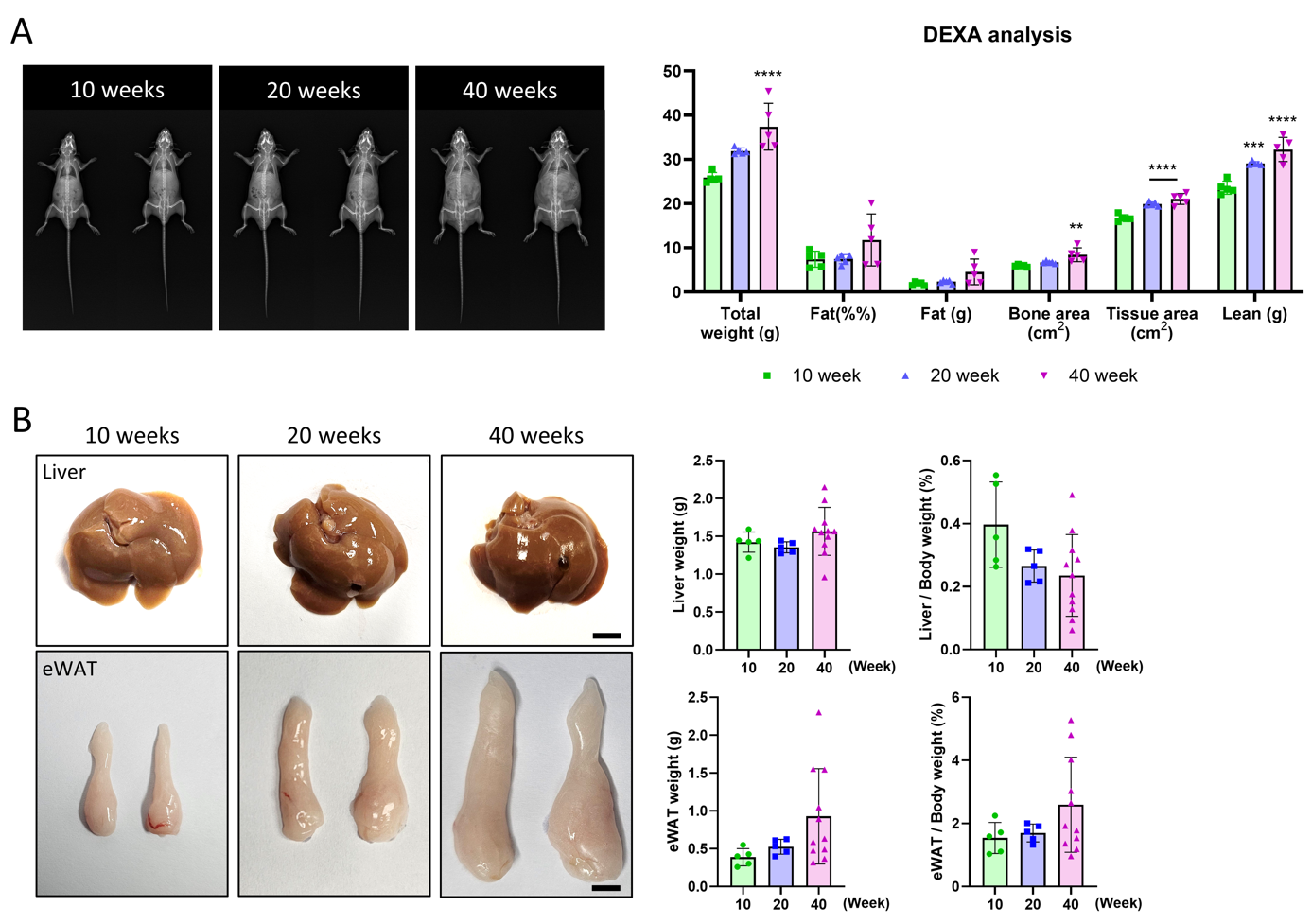


**Figure S8.** Characterizing the atherosclerosis mouse model. (A) Representative DEXA images of *ApoE*^−/−^ mice at 10, 20, and 40–44 weeks (*n* = 5, 5, and 11, respectively), with total mass, fat mass, lean mass, tissue area, and bone area quantified. Significant increases in fat mass and total mass were observed with the progression of atherosclerosis. (B) Representative liver and epididymal white adipose tissue (eWAT) images from *ApoE*^−/−^ mice at 10, 20, and 40–44 weeks (*n* = 5, 5, and 11, respectively). The absolute weights of the liver and eWAT were quantified along with and their weights relative to body weight. Scale bars: 0.5 cm. All values are means ± SDs: ***p* < 0.01; ****p* < 0.001; *****p* < 0.0001 (one-way ANOVA for A and B).


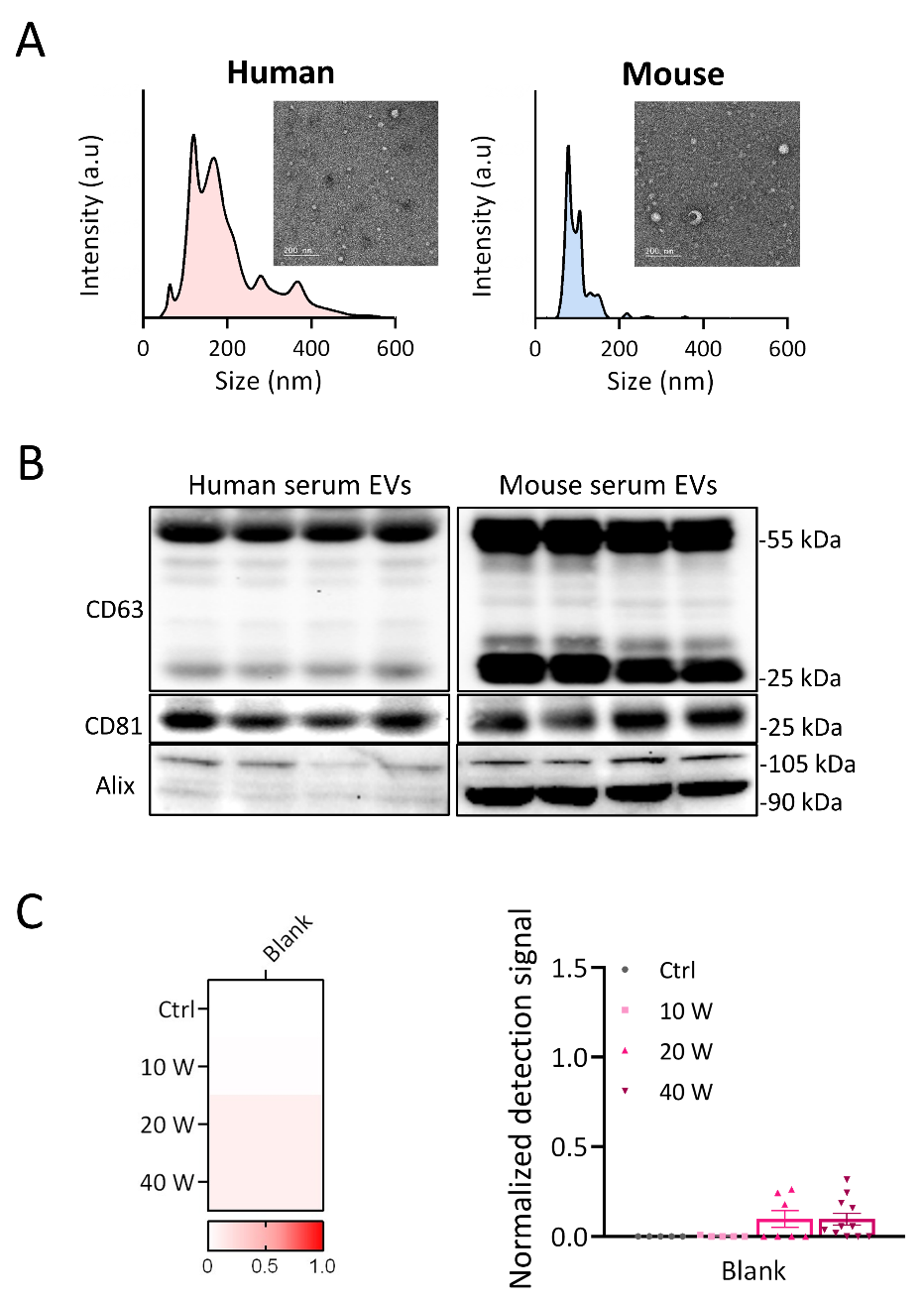


**Figure S9.** Characterization of serum EVs and fRCA reaction for blank sample (A) Size-distribution profile for serum EVs. (A) Size-distribution profile and TEM image for human (left) and mouse (right) serum EVs. Scale bar: 200 nm. (B) Western blot analysis of EV marker proteins (CD63, CD81 and Alix) for human (left) and mouse (right) serum EVs. (C) RCA reaction under the blank condition without the miRNA padlock probe in mouse serum EVs. Each data point represents an individual mouse. W, weeks.
